# Anais Brasileiros de Dermatologia: 2021–2025 Term. Work and Challenges^☆^

**DOI:** 10.1016/j.abd.2021.01.001

**Published:** 2021-02-16

**Authors:** Silvio Alencar Marques, Ana Maria Roselino, Hiram Larangeira de Almeida, Luciana P. Fernandes Abbade

**Affiliations:** a Universidade Estadual Paulista, Botucatu, SP, Brazil; b Universidade de São Paulo, Ribeirão Preto, SP, Brazil; c Universidade Federal de Pelotas e Universidade Católica de Pelotas, Pelotas, RS, Brazil

Dear Colleagues,

We have started our journey with the challenge first of all of maintaining the quality of the hard work of the former Editors and of raising, as much as possible, the scientific quality of *Anais Brasileiros de Dermatologia* (ABD). Historically, ABD has received unrestricted support from the Brazilian Society of Dermatology, from their Board of Directors and administrative staff to the youngest associate. This support is crucial, reflected by the careful and regular reading, constructive criticism and suggestions, the dissemination and appreciation of articles of interest, and by the submission of excellent work.

It is never too much to remember that ABD is the only Dermatology journal in Latin America that is indexed in Medline and, together with *Actas Dermo-Sifiliográficas* of *Academia Española de Dermatología y Venereología*, the only ones in Ibero-Latin America.^1^ This fact translates the dimension of our responsibility and the challenges related to the qualification and representation of Brazilian dermatology, as well as that of our sister countries. The growth of ABD, since its indexing in 2009, can be evaluated in an excellent article by Miot et al.,^2^ which depicts and discusses the ABD bibliographic data related to the period of 2010–2019. We must acknowledge the presented data and we have drawn useful lessons from them to increase the scope and visibility of the Journal.

At present, our intervention is restricted to changing the denomination of the “Investigation” section to “Original Articles”, aiming at expanding it and opening space for case series reports, basic research in Dermatology, clinical trials, epidemiological surveys, and systematic reviews. That does not mean that these topics have not being published in ABD, but we want to make this option clearer. Adjustments to the publication rules will also be implemented, as well as initiatives to facilitate article submission.

An effective intervention regarding the content of the fascicles will start with number 4 of volume 96. Nevertheless, we will continue to place emphasis on Tropical Dermatology, Infectious and Parasitic Dermatoses, STIs and AIDS (open to research, reports and case series) and on Dermatopathology, as these are differential topics and commitments of our Journal, emphasizing that these publications are free of charge for the authors.

Finally, we urge Brazilian and Latin American Dermatologists to make ABD the channel for the dissemination of their studies and experiences, in compliance with the standards and quality of photographic documentation and text review, be it in Portuguese or English. We are awaiting you.

## Financial support

None declared.

## Authors’ contributions

Silvio Alencar Marques: Manuscript preparation and writing.

Ana Maria Roselino: Manuscript preparation and writing.

Hiram Larangeira de Almeida Jr: Manuscript preparation and writing.

Luciana P Fernandes Abbade: Manuscript preparation and writing.

## Conflicts of interest

None declared.

## References

[1] Romani J., Baselga E., Mitjà O., Riera-Martí N., Garbayo P., Vicent A. (2021). Chilblain and acral purpuric lesions in Spain during COVID confinement: retrospective analysis of 12 cases. Actas Dermo-Sifiliográficas.

[2] Miot HA, Ianhez M, Ramos PM. Trends in the main bibliometric indicators of the journal Anais Brasileiros de Dermatologia (2010-2019). An Bras Dermatol. 10.1016/j.abd.2020.11.006. PMC8178526 33775480

